# Supplementary material for: C-reactive protein-based tuberculosis triage testing: a multi-country diagnostic accuracy study
Source: medRxiv. 2024 Apr 24:2024.04.23.24305228. Preprint. [Version 1] doi: 10.1101/2024.04.23.24305228 (PMC11071588; doi:10.1101/2024.04.23.24305228)
Supplement: Supplement 1 [file media-1.pdf]

Supplemental Tables

Table S1. Descriptive characteristics of participants with diabetes.

	Overall N (%)	Uganda	South Africa	Philippines	Vietnam	India
**PWD**	356/2708 (13%)	58/709 (8%)	23/424 (5%)	72/582 (12%)	107/544 (20%)	96/449 (21%)
**Prior diabetes diagnosis**	215 (60%)	15 (26%)	10 (44%)	46 (64%)	71 (66%)	73 (76%)
**On insulin**	24 (11%)	2 (13%)	3 (30%)	1 (2%)	11 (16%)	7 (10%)
**HbA1c (%), median[a]**	6.9 (6.4, 8.6)	6.7 (6.5, 7.3)	6.7 (6, 7.1)	7.2 (6.1, 10.1)	6.8 (6.2, 9.2)	7.2 (6.5, 8.8)
**HbA1c ≥7.0%**	170/352 (48%)	23/58 (40%)	9/22 (41%)	40/72 (56%)	46/107(43%)	52/93 (56%)

Abbreviations: PWD – people with diabetes, HbA1c – hemoglobin A1c.

Footnote: [a]Four participants were missing HbA1c result.

Table S2. Diagnostic accuracy of CRP (5 mg/L cut-point) in reference to MRS, by region.

	Africa N=1227	Asia N=1677	Difference (95% CI)	P-value for difference
**Sensitivity n/N (95% CI)**	303/331 92% (88-94%)	213/282 76% (70-80%)	-16% (-22 to -10%)	<0.01
**Specificity n/N (95% CI)**	441/896 49% (46-53%)	966/1395 69% (67-72%)	+20% (16 to 24%)	<0.01
**PPV n/N (95% CI)**	303/758 40% (37-44%)	213/642 33% (30-37%)	-7% (-12 to 2%)	<0.01
**NPV n/N (95% CI)**	441/469 94% (92-96%)	966/1035 93% (92-95%)	-1% (-3 to 2%)	0.61

Abbreviations: CRP – C-reactive protein, MRS – microbiological reference standard, CI – confidence interval, PPV – positive predictive value, NPV – negative predictive value.

Table S3. Diagnostic accuracy of CRP (5 mg/L cut-point) in reference to sputum Xpert MTB/RIF Ultra results, overall study population and by country.

	Sensitivity (95% CI)	Specificity (95% CI)	PPV (95% CI)	NPV (95% CI)
**Overall**	487/562<br>87% (84-89%)	1423/2325<br>61% (59-63%)	487/1389<br>35% (33-38%)	1423/1498<br>95% (94-96%)
**Uganda**	211/230<br>92% (87-95%)	280/484<br>58% (53-62%)	211/415<br>51% (46-56%)	280/299<br>94% (90-96%)
**South Africa**	79/81<br>98% (91-100%)	166/424<br>39% (35-44%)	79/337<br>23% (19-28%)	166/168<br>99% (96-100%)
**Philippines**	36/51<br>71% (56-83%)	423/542<br>78% (74-82%)	36/155<br>23% (17-31%)	423/438<br>97% (94-98%)
**Vietnam**	126/157<br>80% (73-86%)	263/402<br>65% (61-70%)	126/265<br>48% (41-54%)	263/294<br>90% (85-93%)
**India**	35/43<br>81% (67-92%)	291/473<br>62% (57-66%)	35/217<br>16% (12-22%)	291/299<br>97% (95-99%)

Abbreviations: CRP – C-reactive protein, CI – confidence interval, PPV – positive predictive value, NPV – negative predictive value.

Table S4. Comparison of sub-group AUCs using DeLong's method.

Sub-group	AUC (95% CI)	p-value
**PWH**	0.77 (0.71, 0.83)	0.10
**People without HIV**	0.82 (0.80, 0.84)	
**PWD**	0.82 (0.77, 0.86)	0.76
**People without diabetes**	0.81 (0.79, 0.83)	
**Male**	0.81 (0.79, 0.84)	0.27
**Female** [a]	0.79 (0.75, 0.83)	

Abbreviations: AUC – area under the curve, CI – confidence interval, PWH – people living with HIV, PWD – people living with diabetes.

Footnote: [a]Two participants declined to state sex at birth.

Table S5. Diagnostic accuracy of CRP (5 mg/L cut-point) in reference to MRS and SXRS, by HIV status.

	Overall	HIV status [a]			
**In reference to MRS**		**PWH**	**HIV-negative**	**Difference (95% CI)**	**p-value**
**Sensitivity n/N (95% CI)**	516/613<br>84% (81-87%)	74/85<br>87% (78-93%)	441/526<br>84% (80-87%)	-3%<br>(-11 to 5%)	0.45
**Specificity n/N (95% CI)**	1407/2291<br>61% (59-63%)	140/312<br>45% (39-51%)	1260/1962<br>64% (62-66%)	+19%<br>(13 to 25%)	<0.01
**In reference to SXRS**					
**Sensitivity n/N (95% CI)**	487/562<br>87% (84-89%)	64/71<br>90% (81-96%)	423/490<br>86% (83-89%)	-4%<br>(-11 to 4%)	0.38
**Specificity n/N (95% CI)**	1423/2325<br>61% (59-63%)	143/321<br>45% (39-50%)	1273/1987<br>64% (62-66%)	+19%<br>(14 to 25%)	<0.01

Abbreviations: CRP – C-reactive protein, MRS – microbiological reference standard, SXRS – sputum Xpert reference standard, CI – confidence interval, PWH – people living with HIV.

Footnote:
[a]19 participants had an unknown HIV status.
TB prevalence among PWH and people without HIV was 21% (p=0.90).

Table S6. Diagnostic accuracy of CRP (5 mg/L cut-point) in reference to MRS and SXRS, by diabetes status.

	Overall	Diabetes status			
**In reference to MRS**		**PWD**	**Non-diabetic**	**Difference**	**p-value**
**Sensitivity n/N (95% CI)**	516/613<br>84% (81-87%)	98/111<br>88% (81-94%)	418/502<br>83% (80-86%)	-5%<br>(-12 to 2%)	0.19
**Specificity n/N (95% CI)**	1407/2291<br>61% (59-63%)	158/271<br>58% (52-64%)	1249/2020<br>62% (60-64%)	+4%<br>(-3 to 10%)	0.26
**In reference to SXRS**					
**Sensitivity n/N (95% CI)**	487/562<br>87% (84-89%)	91/102<br>89% (82-95%)	396/460<br>86% (83-89%)	-3%<br>(-10 to 4%)	0.40
**Specificity n/N (95% CI)**	1423/2325<br>61% (59-63%)	159/277<br>57% (51-63%)	1264/2048<br>62% (60-64%)	+5%<br>(-2 to 11%)	0.17

Abbreviations: CRP – C-reactive protein, MRS – microbiological reference standard, SXRS – sputum Xpert reference standard, CI – confidence interval, PWD – people living with diabetes.

Footnote: TB prevalence among PWD was 29% vs. 20% among people without diabetes (p<0.01).

Table S7. Diagnostic accuracy of CRP (5 mg/L cut-point) in reference to MRS and SXRS, by sex.

	Overall	Sex[a]			
**In reference to MRS**		**Female**	**Male**	**Difference (95% CI)**	**p-value**
**Sensitivity n/N (95% CI)**	516/613<br>84% (81-87%)	144/183<br>79% (72-84%)	372/430<br>87% (83-90%)	+8%<br>(1 to 15%)	0.02
**Specificity n/N (95% CI)**	1407/2291<br>61% (59-63%)	697/1125<br>62% (59-65%)	709/1164<br>61% (58-64%)	-1%<br>(-5 to 3%)	0.61
**In reference to SXRS**					
**Sensitivity n/N (95% CI)**	487/562<br>87% (84-89%)	137/165<br>83% (76-88%)	350/397<br>88% (85-91%)	+5%<br>(-1 to 12%)	0.10
**Specificity n/N (95% CI)**	1423/2325<br>61% (59-63%)	706/1138<br>62% (59-65%)	716/1185<br>60% (58-63%)	-2%<br>(-6 to 2%)	0.42

Abbreviations: CRP – C-reactive protein, MRS – microbiological reference standard,
SXRS – sputum Xpert reference standard, CI – confidence interval, PWD – people living with diabetes.

Footnote:
[a]Two participants declined to state sex at birth.

Table S8. Diagnostic accuracy of CRP (5 mg/L cut-point) in reference to MRS and SXRS, among diabetic sub-groups.

	Sensitivity n/N (95% CI)		Specificity n/N (95% CI)	
	In reference to MRS	In reference to SXRS	In reference to MRS	In reference to SXRS
**All PWD**	98/111<br>88% (81-94%)	91/102<br>89% (82-95%)	158/271<br>58% (52-64%)	159/277<br>57% (51-63%)
**HbA1c ≥7.0%**	52/63<br>83% (71-91%)	49/58<br>85% (73-93%)	71/125<br>57% (48-66%)	72/128<br>56% (47-65%)
**HbA1c <7.0%**	44/46<br>96% (85-100%)	40/42<br>95% (84-99%)	85/144<br>59% (51-67%)	85/147<br>58% (49-66%)
**Previously diagnosed**	51/61<br>84% (72-92%)	48/56<br>86% (74-94%)	115/172<br>67% (59-74%)	117/176<br>67% (59-73%)
**Newly diagnosed[a]**	47/50<br>94% (84-99%)	43/46<br>94% (82-99%)	43/99<br>43% (34-54%)	42/101<br>42% (32-52%)

Abbreviations: CRP – C-reactive protein, MRS – microbiological reference standard, SXRS – sputum Xpert reference standard, PWD – people with diabetes, HbA1c – hemoglobin A1c.

Footnote:
[a]Defined as HbA1c ≥6.5% and no prior reported history.

Table S9. Univariate and adjusted ROC regressions for CRP in reference to MRS.

	Univariate	Multivariate			
	Coefficient (95% CI)	Coefficient (95% CI)	SE	z	P>z
**Female**	-0.093 (-0.27, 0.087)	-0.066 (-0.26, 0.13)	0.10	-0.68	0.50
**Age**	-0.012 (-0.017, -0.006)	-0.0026 (-0.009, 0.004)	0.0033	-0.80	0.42
**Uganda**	--	--			
**South Africa**	0.066 (-0.19, 0.32)	0.42 (0.13, 0.71)	0.15	2.87	0.004
**Philippines**	-0.66 (-0.96, -0.37)	-0.38 (-0.71, -0.040)	0.17	-2.19	0.028
**Vietnam**	-0.71 (-0.93, -0.50)	-0.32 (-0.60, -0.047)	0.14	-2.30	0.022
**India**	-0.48 (-0.81, -0.15)	0.010 (-0.36, 0.38)	0.19	0.05	0.96
**Prior TB history**	-0.31 (-0.52, -0.093)	-0.22 (-0.45, 0.010)	0.12	-1.87	0.061
**HIV-negative**	--	--			
**PWH CD4>200**	0.022 (-0.29, 0.33)	-0.12 (-0.46, 0.22)	0.17	-0.70	0.49
**PWH CD4≤200**	0.49 (0.11, 0.86)	0.43 (0.021, 0.84)	0.21	2.06	0.039
**BMI**	-0.061 (-0.086, -0.037)	-0.044 (-0.070, -0.018)	0.013	-3.32	0.001
**No weight loss or weight loss ≤5kg**	--	--			
**Weight loss >5kg**	0.41 (0.23, 0.58)	0.049 (-0.16, 0.26)	0.11	0.46	0.65
**Heart rate**	0.0098 (0.005, 0.015)	0.0074 (0.002, 0.013)	0.0027	2.76	0.006
**Xpert-Negative/Very Low/Low**	--	--			
**Xpert-Medium/High**	0.62 (0.45, 0.78)	0.44 (0.27, 0.62)	0.089	5.00	<0.001
Constant (intercept)		*0.82 (0.019, 1.62)*	*0.41*	2.01	*0.045*
Constant (slope)		*0.71 (0.66, 0.75)*	*0.023*	*30.87*	*<0.001*

Abbreviations: ROC – receiver operating characteristic, CRP – C-reactive protein, MRS – microbiological reference standard, CI – confidence interval, SE – standard error, TB – tuberculosis, PWH – people living with HIV, BMI – body mass index.

Footnote: Variables that reflected a systemic inflammatory response were not included in the model (e.g. temperature, fever, night sweats).

Table S10. Univariable and multivariable adjusted tobit regressions for elevated CRP on the logarithmic scale.

	Univariable (unadjusted)	Multivariable (adjusted for others in the model)		
	Coefficient (95% CI)	Coefficient (95% CI)	SE	p-value
**Female**	-0.74 (-0.92, -0.57)	-0.40 (-0.55, -0.25)	0.078	<0.001
**Age**	-0.015 (-0.020, -0.009)	-0.0016 (-0.007, -0.004)	-0.002	0.56
**Uganda**	--	--	--	--
**South Africa**	-0.25 (-0.48, -0.015)	0.85 (0.62, 1.08)	0.12	<0.001
**Philippines**	-2.24 (-2.53, -1.95)	-1.22 (-1.49, -0.94)	0.14	<0.001
**Vietnam**	-0.89 (-1.13, -0.65)	-0.57 (-0.81, -0.33)	0.12	<0.001
**India**	-1.43 (-1.68, -1.17)	-0.70 (-0.95, -0.45)	0.13	<0.001
**Prior TB history**	0.24 (0.024, 0.45)	0.080 (-0.099, 0.26)	0.091	0.38
**HIV-negative**	--	--		
**PWH CD4>200**	0.47 (0.19, 0.75)	-0.12 (-0.36, 0.13)	0.13	0.35
**PWH CD4≤200**	1.88 (1.45, 2.30)	0.54 (0.19, 0.88)	0.18	0.002
**Diabetes**	0.31 (0.060, 0.57)	0.53 (0.32, 0.75)	0.11	<0.001
**BMI**	-0.12 (-0.14, -0.10)	-0.075 (-0.091, -0.059)	0.008	<0.001
**No weight loss or weight loss ≤5kg**	--			
**Weight loss >5kg**	1.55 (1.36, 1.73)	0.62 (0.44, 0.79)	0.089	<0.001
**Heart rate**	0.044 (0.039, 0.049)	0.035 (0.030, 0.040)	0.002	<0.001
**Smoked today**	-0.39 (-0.66, -0.12)	-0.61 (-0.85, -0.38)	0.12	<0.001

Abbreviations: CRP – C-reactive protein, CI – confidence interval, SE – standard error, TB – tuberculosis, PWH – people living with HIV, BMI – body mass index.
